# Surgery

**Published:** 1879-05

**Authors:** 


*Collaborators:*

DR. H. GRADLE, DR. L. W. CASE, DR. R. PARK,
DR. R. TILLEY, DR. D. R. BROWER.

### SURGERY.

CASE OF TETANUS CURED BY HYPODERMIC INJECTIONS OF MORPHIA AND ENEMAS OF HYDRATE OF CHLORAL.—(*Journ. de Méd.*, January, 1879.)

The patient entered Necker hospital, in the service of M. Broca, July 19, 1878. She had a voluminous osteo-sarcoma of the upper extremity of the right humerus, which began to show itself four months since. July 24, disarticulation of the shoulder, justified by the state of the bone. Cotton dressing. The dressing not being well supported, Dr. Monod, who took M. Broca's place, August 1, substituted the antiseptic dressing of Lister; wound in perfectly good condition. The first symptoms of tetanus appeared August 12. Patient complained of sore throat, but nothing peculiar was seen on examining the pharynx and tonsils. Jaws slightly closed; some pain on pressing over tonsils, but especially excited when patient swallows. While dressing the wound in the evening patient had a chill. From this time the following treatment was instituted in anticipation of tetanus, of which these appeared to be the first symptoms. An enema containing 4 gms. of hydrate of chloral was given at nignt, and hypodermic injections of morphine during the day. During the treatment which lasted a month, there was persistence of the contraction of the masseter and pharyngeal muscles of variable degree; two or three times the muscles of the back of the neck presented slight contracture, and once those of the back became sensible. Morphine was given according to the indications in these changes. An exaggerated sensibility of the wound, and even proud flesh, was seen during the course of these accidents. The digestive functions were not disturbed, the patient being supported with liquid or finely divided food. The patient was kept in a nearly continuous sleep for nearly a month, the morphine being given not regularly, but as indicated. The temperature never was above 38°6. The cure was complete. Two months after there was a recurrence of the tumor.

